# Notch 1 Is Involved in CD4^+^ T Cell Differentiation Into Th1 Subtype During *Helicobacter pylori* Infection

**DOI:** 10.3389/fcimb.2020.575271

**Published:** 2020-11-02

**Authors:** Jinling Xie, Junjie Wen, Chuxi Chen, Meiqun Luo, Bingxin Hu, Danlin Wu, Jianbin Ye, Yanqing Lin, Lijun Ning, Yunshan Ning, Yan Li

**Affiliations:** ^1^School of Laboratory Medicine and Biotechnology, Southern Medical University, Guangzhou, China; ^2^Affiliated Xinhui People's Hospital, Southern Medical University, Jiangmen, China

**Keywords:** *Helicobacter pylori*, NOTCH1, CD4^+^ T cell, Th1 cell, differentiation

## Abstract

*Helicobacter pylori* infection induces CD4^+^ T differentiation cells into IFN-γ-producing Th1 cells. However, the details of mechanism underlying this process remain unclear. Notch signal pathway has been reported to regulate the differentiation of CD4^+^ T cells into Th1 subtype in many Th1-mediated inflammatory disorders but not yet in *H. pylori* infection. In the present study, the mRNA expression pattern of CD4^+^ T cells in *H. pylori*–infected patients differed from that of healthy control using Human Signal Transduction Pathway Finder RT2 Profiler PCR Array, and this alteration was associated with Notch signal pathway, as analyzed by Bioinformation. Quantitative real-time PCR showed that the mRNA expression of Notch1 and its target gene Hes-1 in CD4^+^ T cells of *H. pylori*–infected individuals increased compared with the healthy controls. In addition, the mRNA expression of Th1 master transcription factor T-bet and Th1 signature cytokine IFN-γ was both upregulated in *H. pylori*–infected individuals and positively correlated with Notch1 expression. The increased protein level of Notch1 and IFN-γ were also observed in *H. pylori*–infected individuals confirmed by flow cytometry and ELISA. *In vitro*, inhibition of Notch signaling decreased the mRNA expression of Notch1, Hes-1, T-bet, and IFN-γ, and reduced the protein levels of Notch1 and IFN-γ and the secretion of IFN-γ in CD4^+^ T cells stimulated by *H. pylori*. Collectively, this is the first evidence that Notch1 is upregulated and involved in the differentiation of Th1 cells during *H. pylori* infection, which will facilitate exploiting Notch1 as a therapeutic target for the control of *H. pylori* infection.

## Introduction

*Helicobacter pylori* is a microaerophilic, spiral-shaped gram-negative bacterium that colonizes the stomach in more than 50% of the world's population and causes many *H. pylori*–associated diseases including gastritis, ulcers, and gastric cancer. *H. pylori* infection often induces robust immune response, which does not appear to be sufficient to completely eradicate pathogens and causes life-long persistent infection. The development of drugs provides powerful tools for controlling infection. However, drug resistance, and refractory and poor compliance remain major problems in *H. pylori*–infected individuals. Thus, there is a need to better understand the pathogenesis of *H. pylori* infection and to explore novel therapeutic strategies for its eradication.

A great body of evidence has proved that CD4^+^ T cells are activated in the acquired immune response against *H. pylori* infection, especially IFN-γ-producing Th1 cells (D'Elios et al., 1997a,b, 2003; Bamford et al., 1998; Mattapallil et al., 2000; Akhiani et al., 2002; Eaton et al., 2006; Sayi et al., 2009; McColl, 2010; Flach et al., 2011; Chen et al., 2013; Gray et al., 2013; Yang et al., 2013; Li et al., 2015, 2016). For example, Th1 cells infiltrated in the human stomach during *H. pylori* infection (Bamford et al., 1998). Either vaccine-induced or host natural protective immunity to *H. pylori* infection depends on Th1-dependent cellular immune response in mice (Ermak et al., 1998; Akhiani et al., 2002; Eaton et al., 2006). A similar predominant Th1 response was observed early in rhesus macaques during acute *H. pylori* infection (Mattapallil et al., 2000). Several research groups including our team demonstrated that immunodominant CD4^+^ epitopes of some *H. pylori* antigens induced a Th1-skewed response in humans (Chen et al., 2013; Yang et al., 2013; Ning et al., 2018). Taken together, these results highlight a vigorous Th1 immune response against *H. pylori* infection. However, the mechanism underlying this process remains elusive.

CD4^+^ T helper cells are critical for the acquired immune responses to combat pathogens by differentiating into a variety of effector cells, including Th1, Th2, Th17, and Treg. Each of the Th cell subsets expresses master transcription factors and produces signature cytokines. Th1 cells secrete the transcription factor T-bet, and the hallmark cytokine IFN-γ is essential for the differentiation. The cytokine milieu generated by activated CD4^+^ T cells themselves is one of the crucial determinants for fate decision into effector Th subtypes during differentiation (Zhu et al., 2010; Schmitt and Ueno, 2015), but the mechanism of action of cytokine cannot fully explain the pathogen-induced differentiation mechanism of CD4^+^ T cells, indicating that other molecules account for this process.

Notch signaling is a conserved pathway that plays an essential role in cell fate determination. There are four Notch receptors (Notch1, 2, 3, 4) and five mammalian ligands (DLL1, DLL3, DLL4, Jagged1, and Jagged2) in mammalian. Once bound by Notch ligands, the receptors release the Notch intracellular domain (NICD) via γ*-*secretase. NICD, the active form of Notch receptor, translocates into the nucleus and activates downstream target genes, such as Hes1. Notch signaling has been considered as a critical regulator for the differentiation and function of immune cells including T cells. There is accumulating evidence that Notch signaling can regulate naive CD4^+^ T-cell differentiation into the Th1 type (Maekawa et al., 2003; Minter et al., 2005; Skokos and Nussenzweig, 2007; Zhang et al., 2011; Roderick et al., 2013; Amsen et al., 2015; Verma et al., 2016; Dua et al., 2019). Studies using preclinical models have also revealed the potential therapy of targeting Notch signaling to reduce immune pathology, highlighting the mechanism by which Notch signaling regulates the differentiation and function of T cell (Amsen et al., 2015). To date, there has been no report on the role of Notch signaling in CD4^+^ T-cell differentiation into Th1 subtypes during *H. pylori* infection. We put forward our hypothesis that Notch signaling might be initiated and activated during *H. pylori* infection that subsequently affects Th1 cell differentiation.

In the present study, the mRNA expression level of Notch1, Hes-1, T-bet, and IFN-γ was upregulated in CD4^+^ T cells from *H. pylori*–infected individuals. T-bet and IFN-γ expression levels were both positively associated with Notch1 expression. *In vitro* inhibition of Notch signaling attenuated Th1 cell response, which may shed light on the use of Notch 1 as a therapeutic target for controlling *H. pylori* infection.

## Materials and Methods

### Study Subjects

The protocol was in conformity with the Institutional Human Ethics Review Board of Clinical Laboratory, the affiliated Xinhui People's Hospital, Southern Medical University, Jiangmen, China. All subjects signed the informed consent and were measured for *H. pylori* infection using C^14^ urea test. At the same time, they were treated under a gastroscope with gastric antrum forceps and detected by rapid urease test. The patients were considered as *H. pylori* positive when the results of two detection methods were both positive. Patients who had received antibiotics or proton pump inhibitors (PPI) within 1–2 weeks before the examination and patients with other serious chronic diseases were excluded. All healthy controls were examined clinically and were not suffering from any infectious disease. Venous blood was collected by venipuncture from *H. pylori*–infected individuals and healthy controls and placed into tubes containing EDTA-K_2_ (BD Vacutainer, New Jersey, USA). Blood samples were analyzed by Sysmex XN2000 Automatic Hematology Analyzer (Sysmex, Tokyo, Japan).

### CD4^+^ T-Cell Isolation

Peripheral blood mononuclear cells (PBMCs) were isolated from *H. pylori*–infected patients and healthy controls using Ficoll-Hypaque density gradient centrifugation (GE Healthcare Bio-Sciences AB, Hamburg, Germany). Then CD4^+^ T cells were purified from PBMCs by human CD4^+^ T-cell magnetic beads (Miltenyi Biotec, Palo Alto, CA, USA). The purity was >95% by flow cytometry determination.

### RNA Extraction

Total RNA was extracted from CD4^+^ T cells using TRIzol (Takara, Dalian, China) following the manufacturer's protocol and treated with RNase-free DNase (Solarbio, Beijing, China). A cDNA synthesis kit (Vazyme, Nanjing, China) was used for reverse transcription according to the manufacturer's instructions.

### RT^2^ Profiler PCR Array

CD4^+^ cells were harvested from *H. pylori*–infected individuals and healthy controls. Total RNA was isolated and cDNAs were generated using RT2 First Strand Kit (SA Biosciences, Frederick, USA). Human Signal Transduction Pathway Finder PCR Array including the cDNAs of 84 key genes representative of 10 different signal transduction pathways (PAHS-014Z; SA Biosciences) was used to determine the major signal transduction pathways induced by *H. pylori* infection as described by the manufacturer. RT^2^-PCR was conducted with the following conditions: one cycle of 95°C for 10 min, followed by 40 cycles of 95°C for 15 s and 60°C for 1 min in an ABI-7300 Q-PCR System (Applied Bio-systems, Foster, CA, USA). Genomic DNA control, positive PCR control, and reverse transcription control provided by the manufacturer were used as quality control. The data were analyzed by software provided by SA Biosciences.

### Quantitative Real-Time PCR

Quantitative real-time PCR was amplified using SYBR Green PCR mixture (Vazyme) to quantify the levels of Notch1, Hes1, T-bet, and IFN-γ with the following conditions in an Applied Biosystems instrument: 40 cycles of 95°C for 30 s, 95°C for 10 s, and 60°C for 30 s. All experiments were repeated at least thrice independently to ensure the reproducibility. The sequences of primers are listed in Table 1. The relative fold change of mRNA levels of Notch1, Hes1, T-bet, and IFN-γ was determined using the ΔΔCt method (Li et al., 2012). The averages and SDs were calculated from triplicate datasets. ΔCt was the difference between Ct of target gene mRNA and Ct of internal control β-actin.

**Table 1:** Primers for quantitative real-time PCR.

**Name**	**Primer sequences**
Notch1	Forward, 5′-CACTGTGGGCGGGTCC-3′ Reverse, 5′-GTTGTATTGGTTCGGCACCAT-3′
Hes1	Forward, 5′-CGTGTCTCCTCCTCCCATT-3′ Reverse, 5′-GAGAGGTAGACGGGGGATTC-3′
T-bet	Forward, 5′-GGATGCGCCAGGAAGTTTCA-3′ Reverse, 5′-GACTGGAGCACAATCATCTGGG-3′
IFN-γ	Forward, 5′-GTGTGGAGACCATCAAGGAAGACA-3′ Reverse, 5′-TTGGACATTCAAGTCAGTTACC-3′
β-actin	Forward, 5′-TGGCACCCAGCACAATGAA-3′ Reverse, 5′-CTAAGTCATAGTCCGCCTAGAAGCA-3′

### CD4^+^ T-Cell Culture and Stimulation

CD4^+^ T cells were placed into 24-well plates at a density of 1 × 10 ^6^ cells/ml and cultured in RPMI-1640 supplemented with 10% of fetal bovine serum (FBS; Invitrogen) and 1% penicillin/streptomycin (100 U/ml; Invitrogen) at 37°C in a humidified atmosphere of 5% CO_2_. Anti-CD3/CD28 (eBioscience, San Diego, CA, USA; 1 mg/ml) was added for maintenance of CD4^+^ T-cell activity. To inhibit Notch signaling, CD4^+^ T cells were pretreated with 20 μmol/L γ-secretase inhibitor (*N*-[*N*-(3,5-difluorophenacetyl)-1-alanyl]-*S*-phenylglycine *t*-butyl ester, DAPT) (Sigma-Aldrich, St. Louis, MO, USA) or its vehicle dimethyl sulfoxide (DMSO, 0.08%) for 24 h before *H. pylori* stimulation (MOI 50). Cells and supernatants were collected for further analyses.

### Enzyme-Linked Immunosorbent Assay for IFN-γ

The secretion of IFN-γ in the culture supernatant of CD4^+^ T cells was assessed using CUSABIO (CUSABIO Biotech, Wuhan, China) following the manufacturer's instruction.

### *Helicobacter pylori* Culture

*H. pylori* stain SS1 was grown on Columbia ISO agar plates for 2 days at 37°C, and then transferred to *Brucella* broth supplemented with 5% fetal calf serum (FCS) and antibiotics (polymyxin B, 20 U/ml; vancomycin, 10 mg/ml; trimethoprim, 5 mg/ml) and incubated shaking overnight at 37°C under microaerophilic conditions. Colonies were directly taken from plates and resuspended in RPMI-1640 supplemented with 20% FBS without any antibiotics. Bacteria density was estimated spectrophotometrically. Bacterial count was calculated by determining the optical density (OD) at a wavelength of 600 nm (1 OD = 10^9^ CFU/ml).

### Flow Cytometry

CD4^+^ T cells from *H. pylori*–infected individuals and uninfected controls were assessed in terms of Notch1 and IFN-γ expression by flow cytometry. Briefly, CD4^+^ T cells were fixed, permeabilized, and stained with PE-Notch1 (clone mN1A) and FITC-IFN-γ (clone 4S.B3) antibodies or appropriate isotypes (eBioscience, San Diego, CA, USA). Cells used for flow cytometry were incubated with 7-AAD dye at room temperature for 10 min, and 7-AAD–negative cells were considered as live cells. Flow cytometry was conducted on FACSCalibur flow cytometer (Becton Dickinson, San Jose, CA, USA), and the data were analyzed using CellQuest software (Becton Dickinson).

### Statistical Analysis

Statistical analysis was performed by GraphPad Prism 6.0 software. The results were expressed as the mean ± SD and analyzed by Student's *t*-test. Pearson correlation analysis was applied to assess the relationship between two groups. Differences were considered to be statistically significant when the *p*-value was < 0.05. ^*^p < 0.05; ^**^p < 0.01; ^***^p < 0.001; ^****^p < 0.0001.

## Results

### The Number of Lymphocytes in Peripheral Blood of *Helicobacter pylori*–Infected Patients Was Lower Than Those of Healthy Controls

A total of 91 participants were enrolled in this study. Among them, 52 subjects were confirmed as *H. pylori* positive, and 39 individuals served as healthy controls. The main characteristics of *H. pylori*–infected individuals are listed in Table 2. No significant difference was observed in gender and age between *two* groups. The absolute number of white blood cells, granulocytes, and monocytes was comparable. Only the lymphocytes of *H. pylori*–infected individuals were significantly lower than those of healthy controls.

**Table 2:** Characteristics of *Helicobacter pylori*–infected subjects and healthy controls enrolled in this study.

**Characteristics**	***H. pylori*^**+**^**	**Control**	***P*-value**
Numbers	52	39	
Sex, males/females	35/17	23/16	
Age, mean (x ± s)	58.79 ± 1.78	54.21 ± 2.38	0.12
White blood cells (E + 09/L)	6.96 ± 0.26	7.16 ± 0.26	0.61
Granulocytes (E + 09/L)	4.32 ± 0.25	3.92 ± 0.20	0.23
Lymphocyte (E + 09/L)	2.00 ± 0.08	2.55 ± 0.11	^***^
Monocyte (E + 09/L)	0.44 ± 0.02	0.45 ± 0.02	0.70

*** *p < 0.001*.

### The Notch Signaling in CD4^+^ T Cells of *Helicobacter pylori*–Infected Individuals Was Altered Compared With That of Healthy Controls

**Figure 1:**
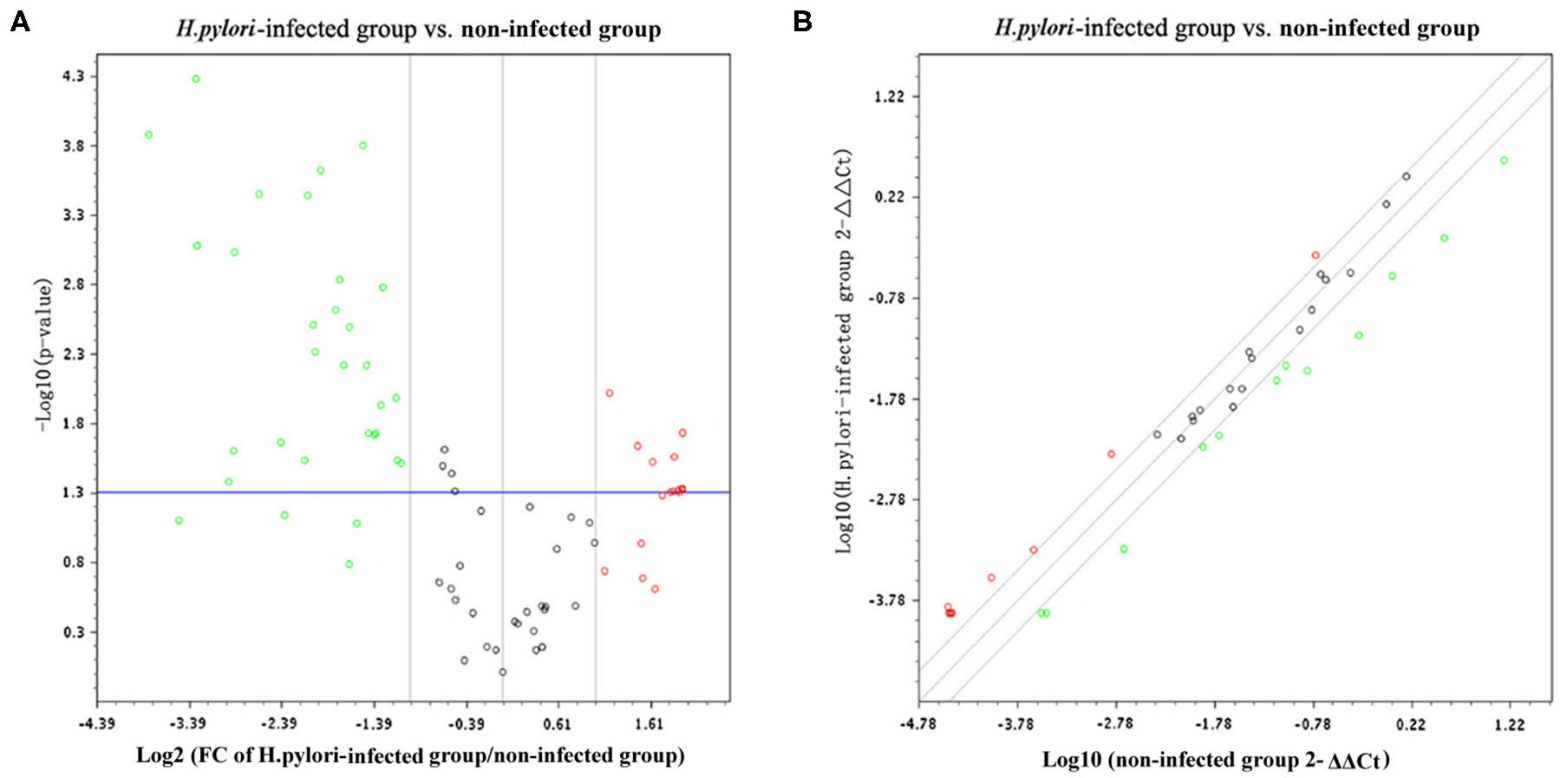
The results of PCR array analysis showing that the signal pathway of *Helicobacter pylori*–infected subjects was altered compared with healthy controls. **(A)** Volcano plot: the red and green circles indicate upregulated and downregulated genes (>2-fold), respectively. The black circles represent no change. The blue line indicates the desired 0.05 threshold for *p*-value of *t*-test. **(B)** Scatter plot. The center line represents genes with no difference in expression. The upper left point represents genes with upregulated expression, and the lower right point represents genes with downregulated expression.

There is a growing body of evidence that Notch signaling can facilitate naive CD4^+^ T-cell differentiation into Th1 subtype (Maekawa et al., 2003; Minter et al., 2005; Skokos and Nussenzweig, 2007; Zhang et al., 2011; Roderick et al., 2013; Amsen et al., 2015; Verma et al., 2016). To explore whether Notch signaling regulates the differentiation of CD4^+^ T cells during *H. pylori* infection, PCR microarray for signal transduction pathways was employed to compare CD4^+^ T-cell mRNA levels between *H. pylori*–infected individuals and healthy controls. It was observed that the signaling pathway of *H. pylori*–infected subjects differed from that of healthy controls. Bioinformatics analysis showed that this alteration was related to various signaling pathways for activating T cells, including Notch signaling (Figure 1).

### Notch1 mRNA Expression Was Elevated in CD4^+^ T Cells of *Helicobacter pylori*–Infected Individuals

**Figure 2:**
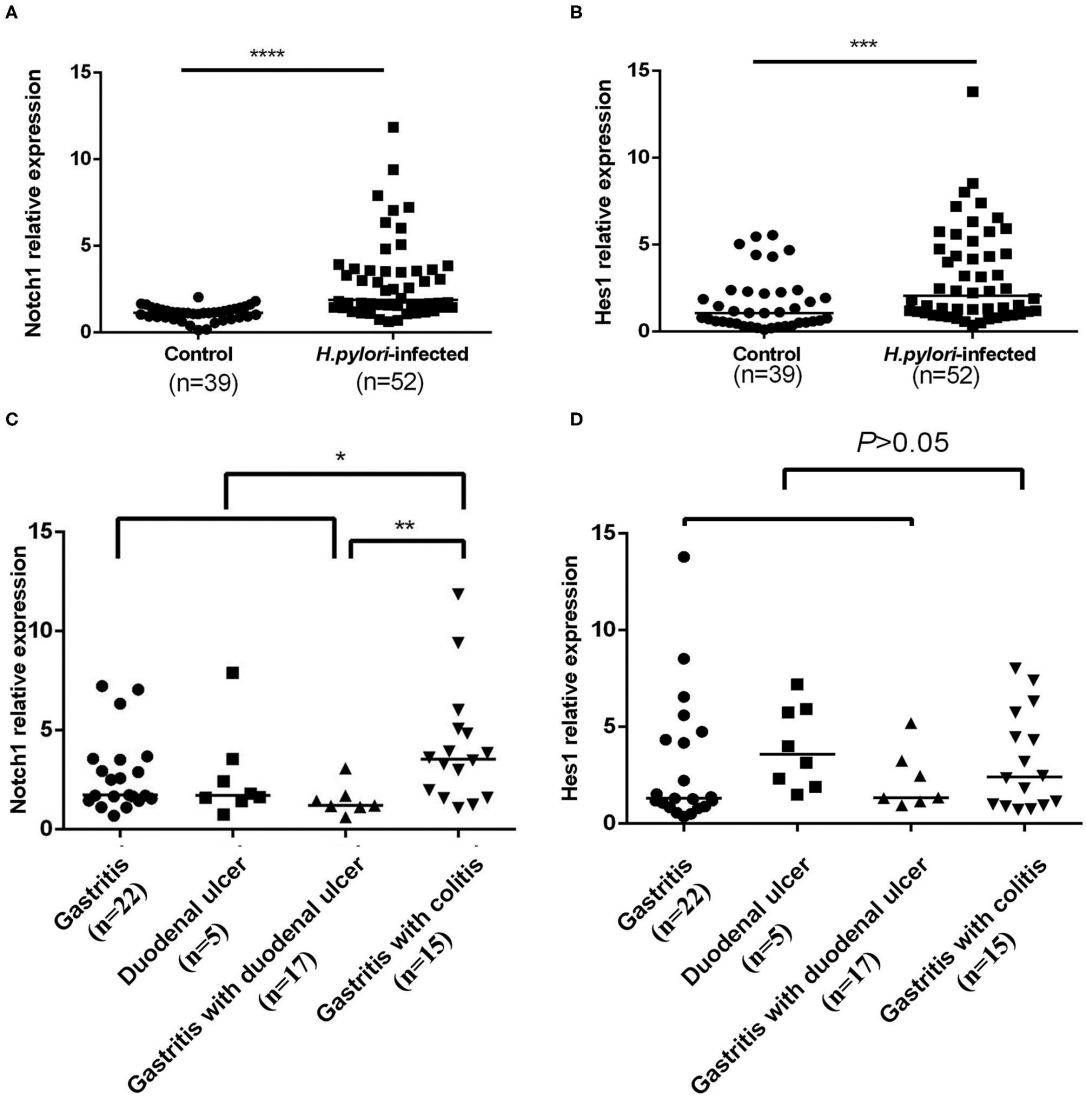
Increase in mRNA expression levels of Notch1 and Hes1 in CD4^+^ T cells of *H. pylori*–infected patients. CD4^+^ T cells were isolated from *H. pylori*–infected subjects (*n* = 52) and the control group (*n* = 39), and Notch 1 and Hes1 mRNA expression was assessed by qPCR. The results were normalized to β-actin. The data are presented as the mean ± SD of three replicates. **(A)** Notch1 expression; **(B)** Hes-1 expression; **(C)** Notch1 expression in different *H. pylori*–associated gastrointestinal disorders; **(D)** Hes1 expression in different *H. pylori*–associated gastrointestinal disorders. *p < 0.05, **p < 0.01, ***p < 0.001, ****p < 0.0001.

Previous studies demonstrated the role of Notch1 in promoting Th1 differentiation (Minter et al., 2005). However, the effect of Notch1 on Th1-mediated response during *H. pylori* infection has not been examined. To investigate whether Notch 1 contributes to *H. pylori* infection, qPCR was applied to compare Notch1 mRNA expression in CD4^+^ T cells between *H. pylori*–infected patients and healthy controls. It was observed that Notch1 expression in the *H. pylori*–infected subjects was significantly higher than that in the control group (Figure 2A). The mRNA level of Hes1, the downstream target of Notch 1, was also upregulated in the *H. pylori*–infected subjects (Figure 2B). In addition, the mRNA expression of Notch1 and Hes1 was assessed in different *H. pylori–*associated gastrointestinal diseases including gastritis, duodenal ulcer, gastritis with duodenal ulcer, and gastritis with colitis. No difference in Notch1 and Hes1 expression was observed among different groups with upper gastrointestinal tract inflammation. However, the expression of Notch1 in the gastritis with colitis group, which involves inflammation of the upper and lower gastrointestinal tract, was significantly higher compared with that in the upper gastrointestinal tract inflammation group. In addition, Notch1 expression in the gastritis with colitis patients was higher than that in the gastritis with duodenal ulcer patients (Figure 2C). However, Hes1 expression between the upper gastrointestinal inflammation group and the colitis group was comparable (Figure 2D). Collectively, these results demonstrate that Notch1 was activated during *H. pylori* infection, and Notch1 mRNA expression was higher when inflammation was more severe.

### T-bet mRNA Expression Was Upregulated in CD4^+^ T Cells of *Helicobacter pylori–*Infected Individuals and Positively Associated With Notch1 Expression

**Figure 3:**
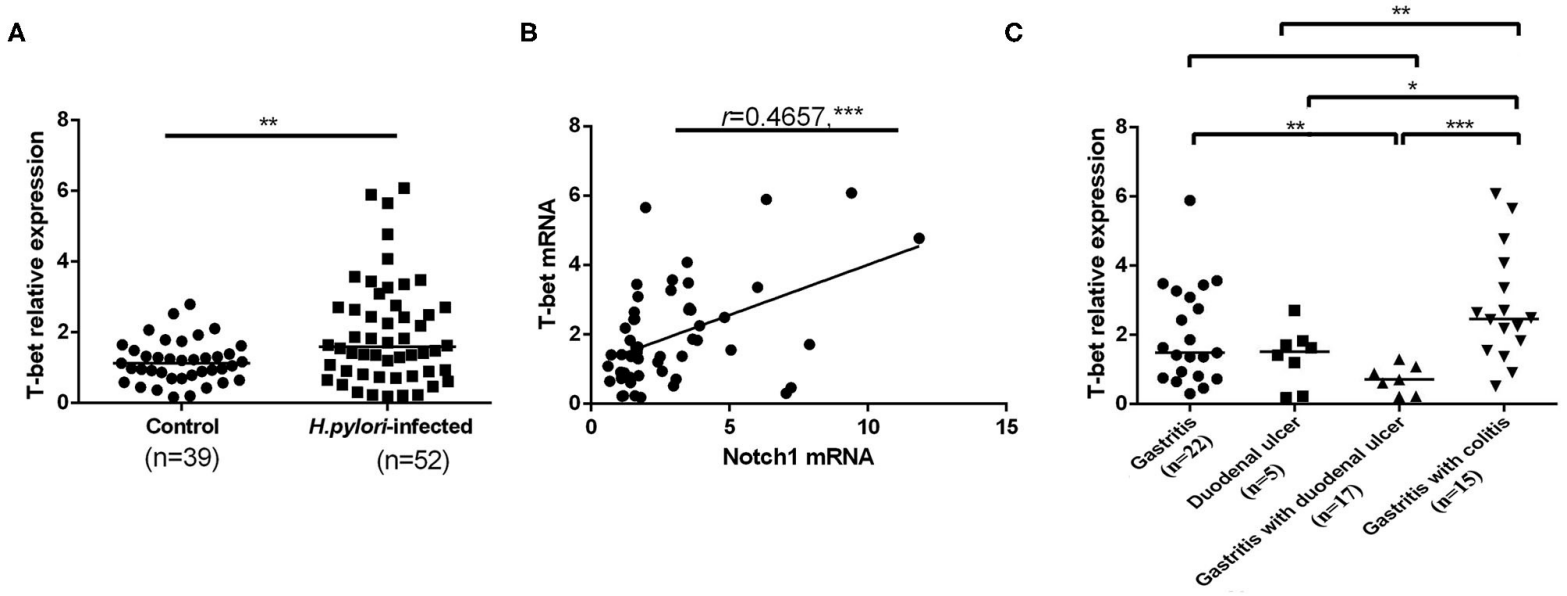
Upregulated mRNA expression of T-bet in CD4^+^ T cells of *H. pylori*–infected patients. CD4^+^ T cells were isolated from *H. pylori*–infected subjects (*n* = 52) and control group (*n* = 39), and T-bet mRNA expression was measured by qPCR. The results were normalized to that of β-actin. The data are expressed as the mean ± SD of three replicates. **(A)** T-bet mRNA expression. **(B)** The correlation of mRNA expression between T-bet and Notch1 was evaluated by Pearson correlation analysis. **(C)** The mRNA expression of T-bet in different *H. pylori*–associated gastrointestinal diseases. *p < 0.05, **p < 0.01, ***p < 0.001.

T-bet is considered as the master transcription factor of Th1 differentiation (Szabo et al., 2002). Previous studies linked Notch signaling to the regulation of Th1 differentiation by activating T-bet (Maekawa et al., 2003; Minter et al., 2005; Tindemans et al., 2017). To further investigate whether Notch1 signaling is involved in Th1 differentiation induced by *H. pylori* infection, T-bet mRNA expression in CD4^+^ T cells from *H. pylor*i–infected individuals was assessed by qPCR. The level of T-bet in *H. pylori*–infected patients was significantly higher than healthy control (Figure 3A). In addition, Pearson correlation analysis revealed that the expression of T-bet in *H. pylori*–infected subjects was positively correlated with Notch1 expression (Figure 3B). Furthermore, T-bet mRNA expression was compared in different *H. pylori–*associated gastrointestinal diseases. When *H. pylori*–infected subjects showed upper gastrointestinal tract inflammation, no significant difference was observed in T-bet expression between the gastritis group and the duodenal ulcer group. Similar results were obtained between duodenal ulcer subjects and gastritis with duodenal ulcer subjects. However, the difference between the gastritis group and the gastritis with duodenal ulcer group was statistically significant. In addition, compared with the upper gastrointestinal tract inflammation group, T-bet mRNA expression in the gastritis with colitis group significantly increased. Moreover, the expression of T-bet in gastritis with colitis subjects was significantly higher than gastritis with duodenal ulcer subjects and duodenal ulcer subjects (Figure 3C). Collectively, these results demonstrate that T-bet is upregulated during *H. pylori* infection and further increases in *H. pylori*–infected patients with more severe inflammation.

### IFN-γ mRNA Expression in CD4^+^ T Cells of *Helicobacter pylori–*Infected Patients Was Upregulated and Positively Correlated With Notch 1 Expression

**Figure 4:**
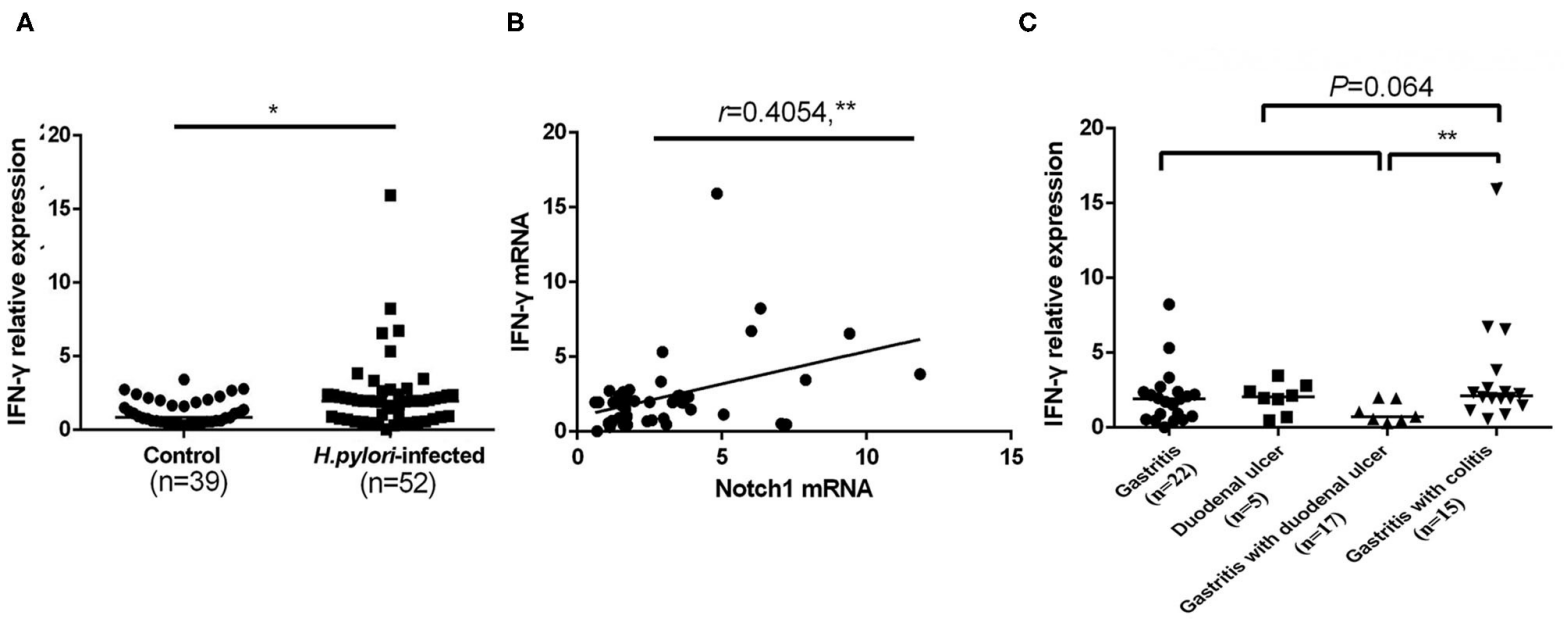
Upregulated mRNA expression of IFN-γ in CD4^+^ T cells of *H. pylori*–infected subjects. CD4^+^ T cells were isolated from *H. pylori*–infected subjects (*n* = 52) and the control group (*n* = 39), and IFN-γ mRNA expression was measured by qPCR. The results were normalized to that of β-actin. The data are presented as the mean ± SD of three replicates. **(A)** The mRNA expression of IFN-γ. **(B)** The correlation between the mRNA expression of IFN-γ and Notch1 was evaluated by Pearson correlation analysis. **(C)** The mRNA expression of IFN-γ in different *H. pylori*–associated gastrointestinal diseases. *p < 0.05, **p < 0.01.

IFN-γ is a hallmark Th1-associated cytokine. Therefore, IFN-γ mRNA expression in CD4^+^ T cells of *H. pylori–*infected subjects was assessed by qPCR. The expression of IFN-γ in *H. pylori*–infected patients was higher than healthy controls (Figure 4A), which was concordant with the results of many previous studies. Pearson correlation analysis showed that the expression of IFN-γ in *H. pylori*–infected patients was positively correlated with Notch1 expression (Figure 4B). Moreover, the expression of IFN-γ was compared in different *H. pylori–*associated gastrointestinal disorders. When *H. pylori*–infected individuals showed upper gastrointestinal tract inflammation, no statistically significant difference was observed among groups. The expression of IFN-γ in the gastritis with colitis group was comparable with the upper gastrointestinal inflammation group. However, the expression of IFN-γ in gastritis with colitis patients was higher than the gastritis with duodena ulcer group (Figure 4C). Overall, these results suggest that IFN-γ increases during *H. pylori* infection, and the expression is associated with Notch1 expression.

### Notch 1 and IFN-γ Protein Levels Were Enhanced in CD4^+^ T Cells of *Helicobacter pylori–*Infected Subjects

**Figure 5:**
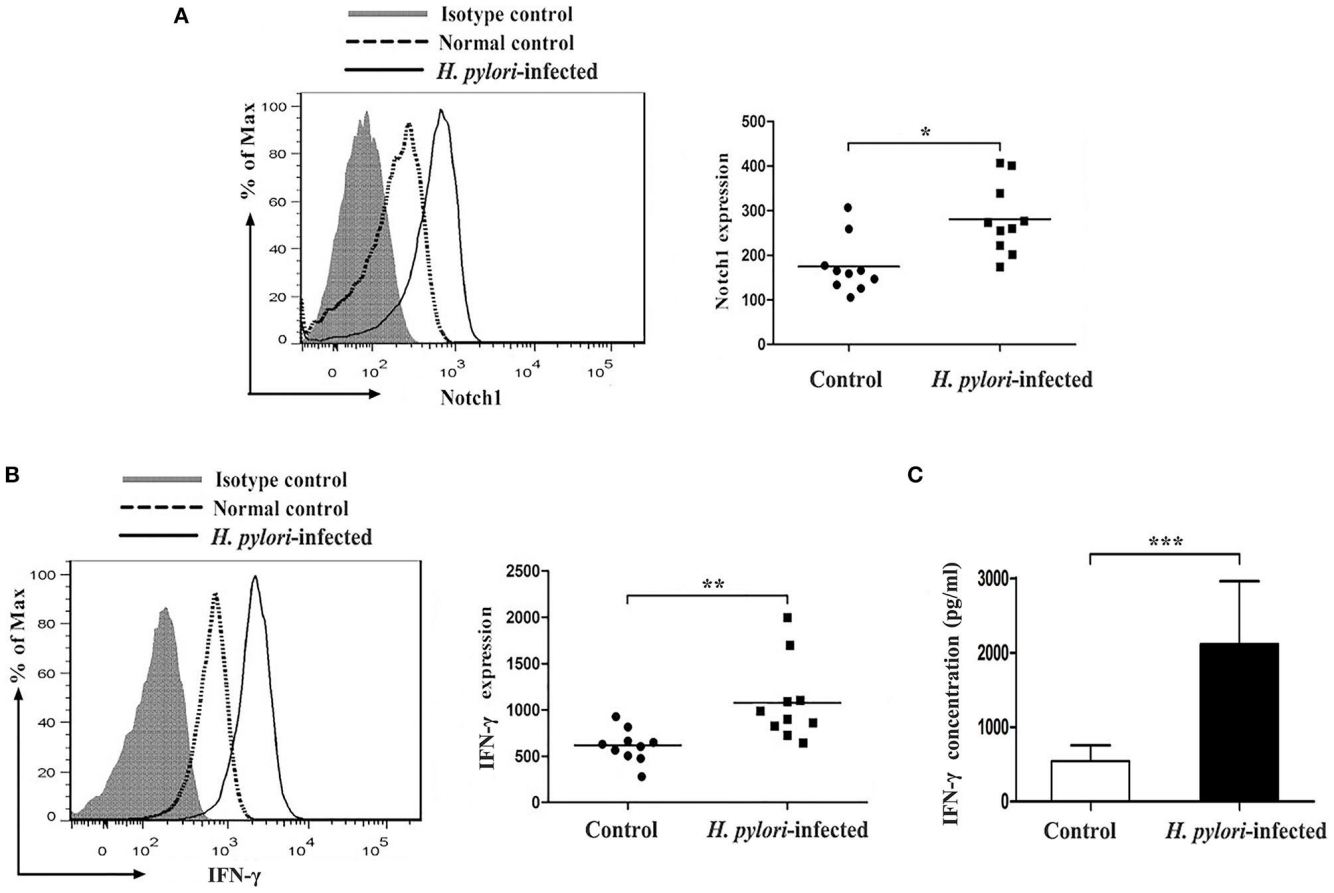
Upregulation of Notch 1 and IFN-γ protein expression in CD4^+^ T cells of *H. pylori*–infected patients. CD4^+^ T cells were isolated from *H. pylori*–infected subjects (*n* = 10) and the control group (*n* = 10) and stained with PE-Notch1 and FITC-IFN-γ antibodies. The protein levels of Notch 1 and IFN-γ were determined by flow cytometry. Cells were stained with an isotype-matched antibody as control. The representative flow cytometric result was shown, and the mean fluorescence intensity (MFI) of Notch 1 **(A)** and IFN-γ **(B)** was evaluated. **(C)** The concentration of IFN-γ in the culture supernatant of CD4^+^ T cells from *H. pylori*–infected subjects (*n* = 10) and healthy controls (*n* = 10) was assessed by ELISA. The data are presented as the mean ± SD of three experiments. *p < 0.05, **p < 0.01, ***p < 0.001.

To further confirm whether the protein levels of Notch 1 and IFN-γ in CD4^+^ T cells were also enhanced in *H. pylori*–infected individuals, coinciding with the observed mRNA level, CD4^+^ T cells from *H. pylori*–infected subjects were stained with antibodies specific to Notch 1 and IFN-γ and assessed by flow cytometry. Notch 1 expression in *H. pylori*–infected subjects was upregulated when compared with healthy controls (Figure 5A). IFN-γ levels also increased in *H. pylori*–infected patients (Figure 5B). Furthermore, ELISA revealed an elevated concentration of IFN-γ in the culture supernatant (Figure 5C).

### Inhibition of Notch Signaling Attenuated the Expression of Notch 1 and Hes1 and the Secretion of IFN-γ Expression *in vitro*

**Figure 6:**
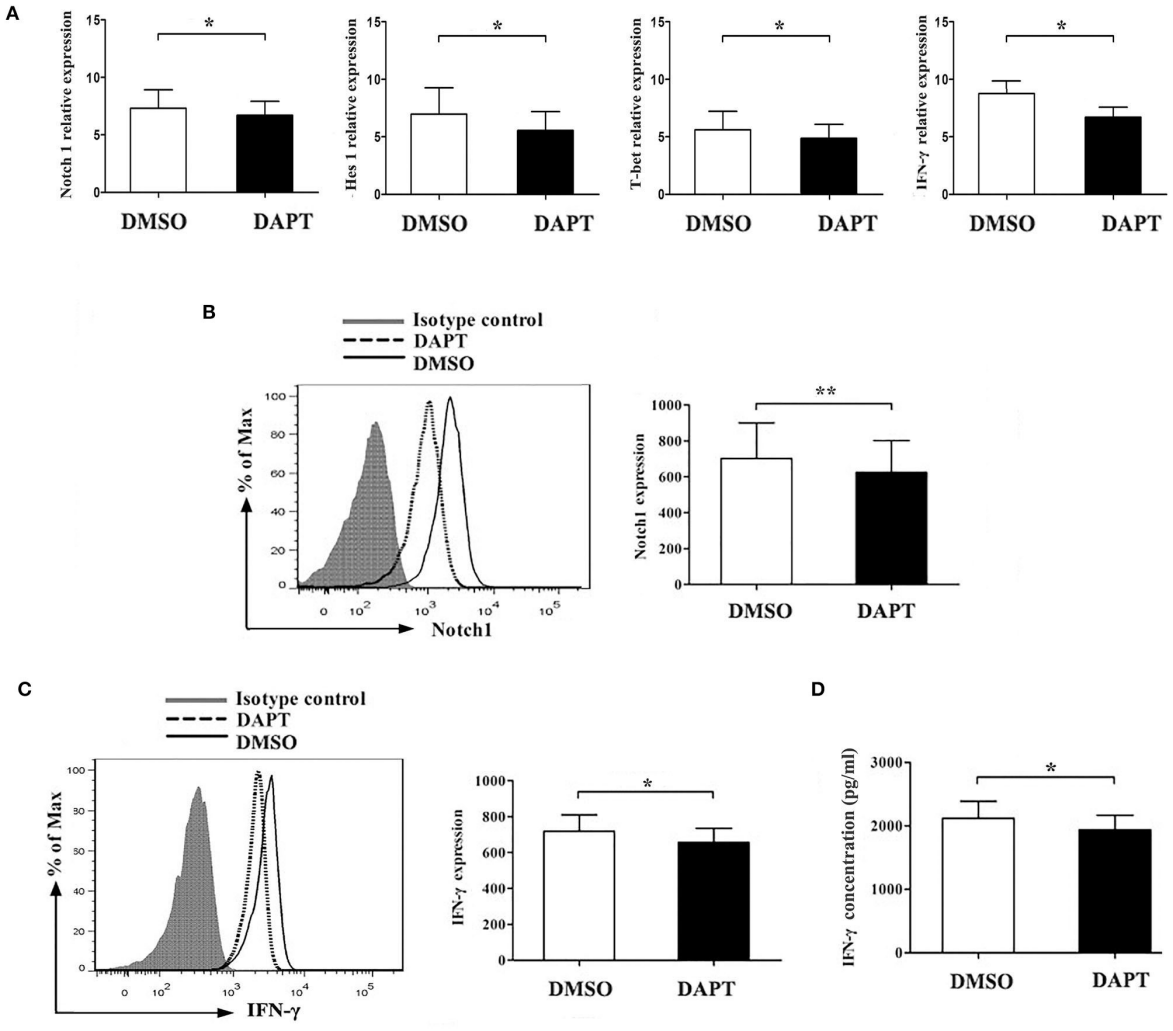
Inhibition of Notch signaling decreases Th1 response in *H. pylori*–infected subjects. CD4^+^ T cells were isolated from *H. pylori*–infected subjects (*n* = 10) and incubated with 20 μmol/L DAPT for 24 h before stimulation with *H. pylori* (MOI = 50). Cells were incubated with DMSO as control. **(A)** The mRNA expression of Notch1, Hes-1, T-bet, and IFN-γ in CD4^+^ T cells of *H. pylori*–infected patients was detected by qPCR. The results were normalized to that of β-actin. The data were presented as the mean ± SD of three replicates. **(B)** Cells were stained with PE-Notch1 antibody with an isotype antibody as control, and the mean fluorescence intensity (MFI) of Notch 1 was evaluated with and without DAPT treatment. **(C)** Cells were stained with FITC-IFN-γ antibody with isotype antibody as control. A representative flow cytometric result was shown, and the MFI of Notch 1 was evaluated with and without DAPT treatment. **(D)** The concentration of IFN-γ in the culture supernatant of CD4^+^ T cells from *H. pylori*–infected subjects (*n* = 10) with and without DAPT treatment was detected by ELISA. The data are presented as the mean ± SD of three experiments. *p < 0.05, **p < 0.01.

Our findings showed that Notch1 signaling was involved in Th1 differentiation of *H. pylori*–infected patients. To explore whether blocking the Notch signaling influences the Th1 response, we treated CD4^+^ T cells with DAPT for 24 h before *H. pylori* stimulation *in vitro*. We observed a decrease in Notch1, Hes-1, T-bet, and IFN-γ mRNA expression (Figure 6A) in association with markedly decreased Notch1 (Figure 6B) and IFN-γ (Figure 6C) protein expression and reduced IFN-γ levels (Figure 6D). These results suggest that the blockage of Notch pathway inhibits Th1 response in *H. pylori*–infected patients.

## Discussion

Cumulative evidence has showed that Notch signaling is involved in CD4^+^ T-cell differentiation and plays a crucial regulatory role (Osborne and Minter, 2007; Minter and Osborne, 2012; Mueller et al., 2013; Laky et al., 2015; Furukawa et al., 2016; Backer et al., 2018), such as involvement in the differentiation of Th subtype (Bailis et al., 2013; Radtke et al., 2013). Of note, many studies have revealed that Notch1 plays a central role in the polarization of CD4^+^ T cells to Th1 subset (Maekawa et al., 2003; Amsen et al., 2007, 2009, 2015; Sun et al., 2008; Mukherjee et al., 2009; Verma et al., 2016; Neal et al., 2017). Notch signaling inhibitors can relieve Th1-mediated model of multiple sclerosis, collagen-induced arthritis (CIA) (Jiao et al., 2014), experimental autoimmune encephalomyelitis (EAE) (Minter et al., 2005), and the symptoms of aplastic anemia (Roderick et al., 2013). Collectively, Notch signaling is involved in Th1 cell differentiation and provides new strategies for the control of several diseases. However, the role for Notch signaling in Th1-mediated *H. pylori*–infected diseases has not been examined. In the present study, the mRNA expression of Notch1 and its downstream target Hes1 in CD4^+^ T cells of *H. pylori*–infected patients was notably higher than that of healthy controls. We also examined the expression of Notch2, Notch3, and Notch4, which did not differ between *H. pylori*–infected patients and healthy controls (data not shown). In accordance with the mRNA levels, the protein expression of Notch1 increased in *H. pylori*-infected subjects. Taken together, elevated Notch1 and Hes1 expression levels indicate an activation of Notch 1 in CD4^+^ T cells from *H. pylori*–infected subjects, which is similar to that of reported Th1-mediated disease. Notch1 expression was stronger during lesion evolution but weaker in the normal tissues in EAE (Seifert et al., 2007). The mRNA expression of Notch1 increased after EAE-specific Ag stimulated peripheral lymphocytes (Jurynczyk et al., 2008). Notch1 expression was higher in *Mycobacterium leprae*–infected patients than in uninfected controls (Dua et al., 2019). The expression of Notch1 also increased in aplastic anemia (Roderick et al., 2013). However, some reports were discordant to the aforementioned results to some degree. The level of Notch1 was significantly lower in SLE patients compared with controls regardless of disease stage. Further, no significant differences were observed in Notch1 expression between patients who took low doses of steroids and those who took moderate doses of steroids (Sodsai et al., 2008).

In addition, the essential role of the Notch signaling has been reported in many inflammation-related diseases (Bassil et al., 2011; Dees et al., 2011; Ishida et al., 2011; Piggott et al., 2011; Gao et al., 2012). Thus, mRNA expression of Notch1 and Hes1 was examined in the CD4^+^ T cells from patients with different *H. pylori–*associated gastrointestinal tract inflammation including gastritis, duodenal ulcer, gastritis with duodenal ulcer, and gastritis with colitis. Notably, the expression of Notch1 was upregulated in the gastritis with colitis group when compared with the upper gastrointestinal tract inflammation group. These results indicate that Notch signal pathway is involved in CD4^+^ T cell immune response to *H. pylori* infection and subsequently affects the clinical outcome.

T-bet is considered to be a master transcription factor for the polarization of CD4^+^ T cells into Th1 and the production of IFN-γ. Several studies have proved that Notch signaling regulates IFN-γ *via* direct regulation of T-bet, and inhibiting Notch signal pathway could decrease IFN-γ secretion in peripheral blood cells (Maekawa et al., 2003; Palaga et al., 2003). CD4^+^ T cells from T-bet^−/−^ mice failed to secrete IFN-γ and thus are limited to non-Th1-subtype responses (Szabo et al., 2002). In aplastic anemia patients, the promoter of TBX21 (T-bet coding gene) binds to NICD1 (the active form of Notch1) to regulate Th1-mediated immune response (Roderick et al., 2013). Given a defined role for T-bet in promoting Th1-mediated cellular immunity, the evidence for Th1-mediated pathology of *H. pylori* infection, and previous observation that Notch1 influences T-bet levels, we wondered whether Notch1 contributes to pathogenesis in *H. pylori*–associated gastrointestinal disease. In our study, the level of T-bet mRNA in *H. pylori*–infected patients was higher than that in healthy controls and was positively correlated with Notch1 expression. Moreover, T-bet further increased in *H. pylori*–infected subjects with more severe inflammation. Taken together, the occurrence of *H. pylori* infection is accompanied by activated Notch1 and elevated expression of T-bet, indicating that Notch1 is a crucial mediator of Th1-mediated pathology in *H. pylori*–associated gastrointestinal disease through direct regulation of T-bet.

IFN-γ is vital in the immunopathogenesis of *H. pylori* infection. Several studies have documented that IFN-γ^−/−^ mice challenged with *H. pylori* did not develop gastritis (Sawai et al., 1999; Smythies et al., 2000; Yamamoto et al., 2004), suggesting that *H. pylori*–induced gastritis is involved in the Th1-mediated response. In our study, it was observed that IFN-γ mRNA expression was upregulated in *H. pylori*–infected subjects and positively correlated with Notch1 expression, suggesting that Notch1 is involved in the differentiation of Th1 cell during *H. pylori* infection. *In vitro*, blockage of Notch signaling decreased IFN-γ expression in CD4^+^ T cells of *H. pylori*–infected patients, which was roughly concordant with previous studies describing that IFN-γ production was inhibited by blockage of Notch signaling in vascular inflammation (Radtke et al., 2010). However, a reduced production of Th2 cytokines in association with an increased production of Th1 cytokines was observed in allergic pulmonary inflammation after inhibiting Notch signaling (Kang et al., 2009). The discrepancies might be attributed to the different immunological mechanisms of various inflammatory disorders.

γ-Secretase inhibitors can successfully block the final enzymatic step required for Notch cleavage and activation and inhibit Notch signaling (Minter et al., 2005). In aplastic anemia, γ-secretase inhibitor reduced T-bet expression and IFN-γ production, thus inhibiting the differentiation of Th1 cells from CD4^+^ T cells *in vitro* and *in vivo* (Roderick et al., 2013). During the process of pulmonary infection induced by *Cryptococcus neoformans*, the secretion of IFN-γ decreased when the Notch signaling was inhibited (Neal et al., 2017). Thus, the application of Notch inhibitors as a therapeutic strategy is of substantial and growing interest. In our study, inhibition of Notch signaling attenuated T-bet expression and IFN-γ secretion of CD4^+^ T cell after stimulation with *H. pylori in vitro*, indicating appropriate Notch blockage may offer novel strategies in reducing *H. pylori*–induced inflammation.

Notch signaling has been considered to play an essential role in the pathogenesis of several clinical diseases, such as rheumatoid arthritis (Gao et al., 2012), systemic sclerosis (Jiao et al., 2010; Dees et al., 2011), and giant cell arteritis (Radtke et al., 2010). Inhibition of Notch signaling attenuated the severity of EAE (Bassil et al., 2011) and experimental autoimmune uveoretinitis (Ishida et al., 2011). It has been acknowledged that inflammation of the gastric mucosa induced by *H. pylori* depends mainly on Th1 cell responses (Smythies et al., 2000; Eaton et al., 2001; Bagheri et al., 2018b). Our study showed for the first time that increased activation of Notch1 was associated with increased Th1 response in *H. pylori*–infected patients, which may contribute to the pathogenesis of *H. pylori*–associated gastroenterological diseases. Most importantly, it is not clear if upregulated Notch signaling represents immune response to infection or drives the associated inflammation, or both. Therefore, care must be taken when proposing to target Notch as a means of controlling inflammation associated with *H. pylori* infection.

In summary, CD4^+^ T cells from *H. pylori*–infected subjects displayed a remarkably elevated Notch1, Hes-1, T-bet, and IFN-γ levels compared with healthy controls, suggesting that increased Notch1 was associated with an enhanced Th1 response during *H. pylori* infection. *In vitro*, Notch signaling inhibitor can efficiently suppress Th1 response. To our knowledge, this is the first evidence that has explored the involvement of Notch signaling in Th1 cell differentiation in *H. pylori* infection.

There are several limitations in the present study. First, previous studies have proved that the number of Th17 cells increased in *H. pylori*–infected patients (Bagheri et al., 2018a), and both Th1 and Th17 may act synergistically to mediate immune response against *H. pylori* and induce mucosal inflammation in *H. pylori*–infected patients (Shi et al., 2010; Bagheri et al., 2018b). Therefore, the expression of Notch1 in Th17 cells in our study should be confirmed to better understand the role of Notch1 during *H. pylori* infection. Second, Notch1 expression should be examined after successful treatment of *H. pylori*–infected patients, which will provide comprehensive data for Notch1 signaling in a longitudinal study. We will continue this study in the future.

## Data Availability Statement

The raw data supporting the conclusions of this article will be made available by the authors, without undue reservation.

## Ethics Statement

The studies involving human participants were reviewed and approved by the Institutional Human Ethics Review Board of Clinical Laboratory, the Affiliated Xinhui People Hospital, Southern Medical University, Jiangmen, China. The patients/participants provided their written informed consent to participate in this study. The animal study was reviewed and approved by the Institutional Human Ethics Review Board of Clinical Laboratory, the Affiliated Xinhui People Hospital, Southern Medical University, Jiangmen, China.

## Author Contributions

JX mainly contributed to the work. JW, CC, ML, BH, DW, JY, and YLin participated in the experiment. YN and YLi designed and contributed to the work, as well as wrote this article. All authors contributed to the article and approved the submitted version.

## Conflict of Interest

The authors declare that the research was conducted in the absence of any commercial or financial relationships that could be construed as a potential conflict of interest.
